# Mental Capacity, Decision-Making and Emotion Dysregulation in Severe Enduring Anorexia Nervosa

**DOI:** 10.3389/fpsyt.2021.545317

**Published:** 2021-03-11

**Authors:** Annemarie van Elburg, Unna Nora Danner, Lot Catharina Sternheim, Mirjam Lammers, Isis Elzakkers

**Affiliations:** ^1^Department of Clinical Psychology, Faculty of Social Sciences, Utrecht University, Utrecht, Netherlands; ^2^Rintveld Center for Eating Disorders, Altrecht Mental Health Institute, Zeist, Netherlands; ^3^GGNet Centre of Mental Health, Apeldoorn, Netherlands

**Keywords:** severe enduring anorexia nervosa, mental capacity, decision-making, intolerance of uncertainty, emotion dysregulation

## Abstract

Severe and Enduring Anorexia Nervosa (SE-AN) is a chronic eating disorder characterized by long-term starvation and its physical and psychological sequelae, and severe loss of quality of life. Interactions between neurobiological changes caused by starvation, vulnerability (personality) traits, and eating behaviors play a role. Several other factors, such as increased fear and decreased social cognition, have also been found in relation to SE-AN. With this in mind, we aim to add to the understanding of SE-AN by introducing the concept of mental capacity (MC), which refers to the ability to understand and process information—both on a cognitive and an emotional level—and then make a well-informed choice. MC may be an important construct within the context of SE-AN. Furthermore, we will argue how impaired decision-making processes may underlie, fuel, or contribute to limited MC in SE-AN. We will speculate on the importance of dysfunctional emotion processing and anxiety-related processes (e.g., a high intolerance of uncertainty) and their potential interaction with decision-making. Lastly, we will propose how these aspects, which to our knowledge have previously received little attention, may advise research and treatment or help in dealing with the “want but cannot” situation of life-threatening AN.

## Introduction

Emma is 39 years old. Her anorexia nervosa (AN) started at age 15. After temporary and partial improvement while she was in treatment during adolescence and early adulthood, her AN shows a slow deteriorating course; she is currently “stable” at a body mass index (BMI) of 13. Emma lives alone, is socially isolated, unemployed, and can barely look after herself. Her whole life revolves around her eating disorder. While it is definitely not her wish to die, she is also unable to change her eating habits in a meaningful way: the “want but cannot” situation so often seen. Her treatment team regards her as suffering from a severe and enduring form of AN.

Emma's (fictitious) case illustrates there seems to be a sense of what constitutes a case of severe and enduring anorexia nervosa (SE-AN). However, SE-AN is not an easy term to define. Robinson (1) introduced the term Severe and Enduring Eating Disorders, comparing the persistence of eating-disorder symptoms to other serious mental illnesses such as schizophrenia. He did not define a clear delineation in time, treatment, or severity of symptoms that marked the severe and enduring character. Attempts have been made to develop a staging model (2), and therapists and patients were asked to provide their views on what constitutes chronic AN. Hay & Touyz (3) proposed criteria (Table 1) based on duration of illness (more than 3 yrs), two or more not successful treatments, and a persistent state of illness with functional impairment but state there are limitations to these criteria that need empirical testing. Overall, no clear-cut picture emerged, although participants did agree on factors relating to weight, behaviors, and cognitions (4). In one qualitative study (5), patients who suffered from AN for over 20 years described that the eating disorder provided meaning and structure, while at the same time it had robbed them of relationships, family, occupation, etc. Patients may express a desire to change but feel incapable and/or unwilling to translate this desire into actual behavior (6), despite the costs to their lives. We found only one model explaining the progress of AN into SE-AN, by Treasure et al. (7) when they revisited their cognitive interpersonal model for AN and specifically looked at perpetuating factors. They describe how AN can develop into a chronic condition through interaction between behavioral consequences (i.e., increased neuroadaptation, food phobia, and habituation), vulnerability (personality) traits (i.e., rigid and anxious temperament), anorectic behaviors, and interpersonal difficulties (increased fear and frustration, alienation, loneliness, and decreased social cognition), combined with chronic stress (increased allostatic load and inflammation, decreased mood and neurogenesis). The model also highlights the role of heightened anxious and depressive symptomatology and dysfunctional emotion processing (such as problems with emotion recognition and regulation). This type of problem in emotional functioning can have debilitating consequences and, hence, can influence functioning on other levels. For example, it is known that emotions and emotion-related processes are essential for the way people make choices and therefore for our decision-making behaviors. Understanding this seems particularly relevant in the clinical situation of AN where the short- and longer-term consequences of a decision do not align and where a variety of emotions is involved.

**Table 1:** Proposed criteria for “Severe and Enduring Anorexia Nervosa” Hay and Touyz (3).

(1) A persistent state of dietary restriction, underweight, and overvaluation of weight/shape with functional impairment
(2) Duration of >3 years of anorexia nervosa; and
(3) Exposure to at least two evidence-based treatments appropriately delivered together with a diagnostic assessment and formulation that incorporates an assessment of the person's eating disorder health literacy and stage of change

This commentary aims to add the concept of mental capacity (MC) to the dialogue about how AN turns into SE-AN. MC refers to the ability to understand and process information both on a cognitive and an emotional level and then make a well-informed choice. MC may be an important construct within the context of SE-AN. We will argue how impaired decision-making processes may underlie, fuel, or contribute to limited MC in SE-AN. We will speculate on the importance of dysfunctional emotion processing and specifically address anxiety-related processes such as a high intolerance of uncertainty (IU) and how they may interact with decision-making. Lastly, we will propose how these findings, which to our knowledge have received little attention until now, may advise treatment or help in dealing with the “want but cannot” situation of life-threatening SE-AN.

## Mental Capacity

When a seriously ill patient refuses a potentially lifesaving intervention, this persons' ability to make an informed decision can be put into question. Clinicians may describe individuals with AN who refuse treatment as having limited MC.

Few studies have been conducted on MC in patients with AN. This is remarkable, as diminished or absent MC is one of the central concepts in the discussion regarding compulsory treatment (CT) (8–16) and the (possible) concept of futility in the treatment of SE-AN (16, 17). MC is a concept that cannot easily be quantified. The way it is conceptualized today derives from legal rulings in the United States in the 1980s. Abilities considered relevant by judges in rulings regarding MC issues were adopted by clinicians in their clinical assessment. The most widely used assessment of MC is the MacCAT-T (18), a semi-structured interview that assesses understanding, reasoning, appreciation, and the ability to express a choice. In the assessment of MC, the clinician assesses the decision-making *process*, rather than the outcome. Since its introduction, the MacCAT-T has emerged as the gold standard in scientific research into MC due to its high interrater reliability, demonstrated concurrent validity with other measures, and extensive testing in a range of patient populations, both medical and psychiatric (13, 19–21). The MacCAT-T was used in two small studies in adolescents with AN (22, 23) and in one larger longitudinal study with severely ill adult patients [mean BMI of 15.5 kg/m^2^, mean length of illness of 8.6 years (24, 25)]. The two adolescent studies showed conflicting findings: one (done retrospectively) not showing problems in MC at all, the other showing mild problems with reasoning. In the longitudinal study, patients with diminished MC seemed to do less well in treatment and displayed more fundamental decision-making deficiencies that did not ameliorate with weight gain. Therefore, diminished MC seems a relevant factor to prognosis, in addition to the more obvious factor of BMI. In this study, the MacCAT-T indicated that it was the aspect of appreciation that was driving diminished MC, in line with the findings by Owen and colleagues (2013) (26) in a general psychiatric population. The concept of appreciation refers to the value patients assign to issues such as the illness itself or the proposed treatment. When appreciating adequately, one for instance feels that the issues discussed apply to oneself (e.g., “I do have an eating disorder” or “This risk applies to me”) and are therefore relevant in the decision-making process. The question emerges *in what way* MC influences prognosis and thus the development of SE-AN. Considering that MC encompasses the decision-making processes in the clinical context and diminished MC is mostly related to distorted appreciation, it is important to understand the role of decision-making in a broader sense.

## Decision-Making

Decision-making processes of people with AN have been the focus of many studies (27–30). Findings indicate that these processes are disturbed as patients' choices seem more guided by the short-term outcomes (e.g., food intake and weight gain) and less by the longer-term outcomes (improved daily functioning) when compared to people without eating disorders or any other form of psychopathology. Decision-making inherently relies on emotional processes that provide important implicit and explicit knowledge by which the individual is able to make fast and adaptive decisions (31). These emotional processes guide decision-making on several levels, including *via* bioregulatory processes, such as somatic marker signals, and occur both consciously and outside of awareness. One hypothesis is that problems in emotional functioning and processes underlying certain emotional experiences, such as uncertainty tolerance, lead to these disturbances.

## Emotional Processing

**Figure 1:**
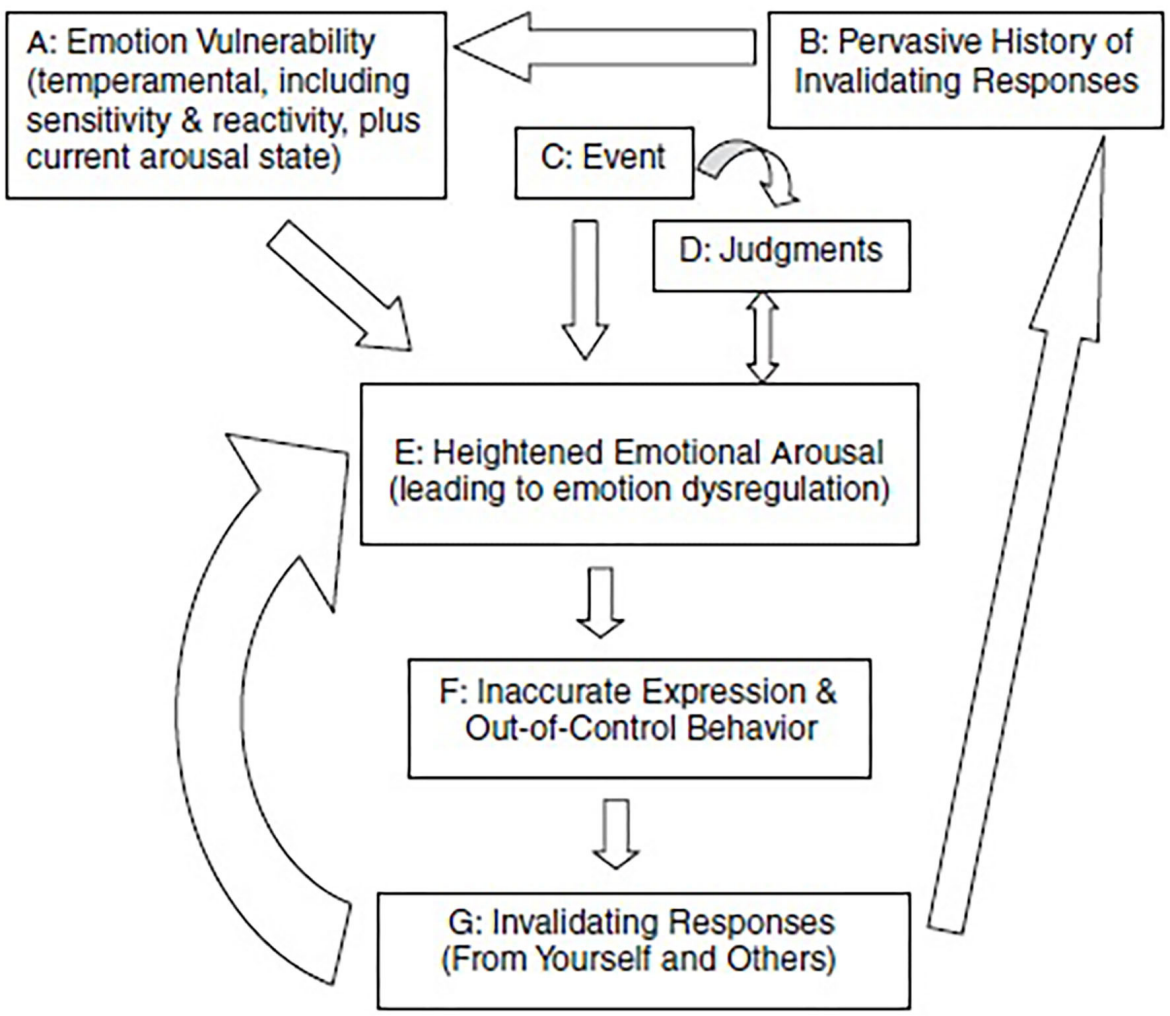
The transactional model of emotion regulation. [Adopted from Haynos and Fruzzetti (33)].

AN has frequently been associated with disturbances in emotions and emotional processing. Even more so, emotion dysregulation is suggested to be at the core of AN (32, 33) (Figure 1). Problematic emotional functioning of people with AN was shown on various levels (33), for example, frequent and intense emotional experiences, problems recognizing and expressing emotions (34–36), lower ability to regulate emotions, and fewer emotion regulation strategies available (32, 37, 38). To complicate matters further, people with AN are suggested to be on average more emotionally sensitive, to experience emotions longer (the feelings and bodily sensations decline less rapidly) and to be less tolerant of emotional experiences than control participants (39, 40). This can result in so-called secondary emotions such as shame, guilt, anxiety, and (feelings of) depression. It is suggested that eating-disorder-related behaviors are used as coping strategies to reduce, distract from, or even numb emotional experiences as other strategies are lacking or do not result in a reduction of the emotions or unpleasant feelings (33, 41, 42). In Figure 1, it reduces the arrow between A and E. A longitudinal study by Racine and Wildes (43) showed that patients with AN who were characterized by high emotion dysregulation reported an increase in AN symptomatology during intensive treatment, and they maintained this high level independent of their low weight over and above their general emotional state. Considering that emotion dysregulation persists with improvement of weight and eating-disorder symptoms, it is regarded as a key factor for relapse and ultimately for chronicity (and thus for the development of SE-AN).

## Affective States and Decision-Making: Intolerance of Uncertainty

Indeed, there is initial evidence that emotional processes, more specifically affective states such as anxiety and depression are associated with poorer decision-making in AN (29, 44). This is not surprising and has been recognized much longer in other clinical research fields [for a review on the relation between affective states and decision-making in anxiety and depression, see Paulus & Angela (45)]. This review highlights that an “affect-driven belief system profoundly influences the transformation of action into choices (p. 477)” and proposes that affect in particular plays a role in decision-making that involves uncertainty, which is similar to the type of decision-making mostly studied in AN (24, 29, 30).

We believe uncertainty intolerance to be a potentially important yet currently undervalued concept in the context of SE-AN. For SE-AN patients, certain eating disorder behaviors may function to reduce precisely this uncertainty and the negative emotions associated to uncertainty [see Sternheim et al. (46)]. Quantitative studies confirm elevated levels of IU in both adolescents and adults with AN (47). IU refers to the fear of the unknown (48), a negative response to uncertain situations on emotional, cognitive, and behavioral levels (49). These findings fit well with the revised model by Treasure at al., which includes rigid and anxious personality traits. IU has been found to contribute to abnormalities in the reward system and subsequent decision-making processes (50). We speculate that training patients in tolerating uncertainty and becoming more flexible and less anxious may improve their general quality of life, probably the most important aspect of treatment in SEAN, and may even result in more adaptive decision-making.

## Discussion

### MC Research

This paper explores the idea that decision-making, and its effect on MC may be important constructs to understand the development of SE-AN. As emotions and emotion regulation play such an important role in decision-making, a focus on these aspects in research regarding MC in AN would be expected. However, the connection to MC in these patients has not yet been thoroughly studied in clinical research, and neither has the relationship between maladaptive decision-making, disturbed emotion processing, and MC. The need to study this relation is supported by the view that MC assessments in general and the MacCAT-T in particular are focusing too much on cognitive and rational functioning, whereas decision-making, as described, is not wholly rational but rather very much influenced by emotional factors (51–55). During a clinical study of the ability to understand and process information both on a cognitive and an emotional level and then make a well-informed choice, we found that it is the aspect of appreciation in MC—the value patients assign to issues such as the illness itself or the proposed treatment—that can become diminished and underlies the “want but cannot” dilemma of the critically ill patient. Intriguingly, the longitudinal study amongst severely ill patients with AN by Elzakkers et al. (25) showed that patients with diminished MC exhibited persistent maladaptive decision-making over the course of a 2-year follow-up even when controlling for BMI, depression, and alexithymia scores (a direct relationship between these emotional problems and maladaptive decision-making was found). This suggests that the difference between full and diminished MC cannot be fully explained by the variation in emotional problems as measured in this study (depression, anxiety, alexithymia). Presumably, other concepts not measured in this study are also important, such as emotion regulation and the ability to tolerate emotions or uncertainty. Interestingly, appreciation ratings (as measured by the MacCAT-T) of the diminished MC group over time remained inferior to the full MC group, even when gaining weight, linking the concept of more fundamental decision-making (and “gut feeling”) to the concept of appreciation in AN. Diminished MC could function as a marker for more severe deficits in decision-making, and underlying disturbed emotional processing, and serve to guide treatment toward implementing a stronger focus on these emotional issues (33, 40).

### MC Treatment

On the basis of these findings, we believe clinicians ought to pay more attention to their patient's current MC, especially in SE-AN, when important treatment decisions are to be made. Diminished MC has grave legal consequences in most medico-legal systems and lessens the say patients have regarding their own treatment legally. Even so, we would like to point out that shared decision-making is not by definition impossible in this situation and in fact may help to discuss how to improve their quality of life. Taking patients seriously in their suffering and anxiety and acknowledging their views is still paramount and should remain one of the pillars in the clinical decision-making.

## Decision-Making and Dysfunctional Emotional Processing Research

Next, we discuss the possibility of underlying dysfunctional emotion processing, such as a high IU, and how this may interact with decision-making (56, 57). A study in healthy individuals and in people with anxiety shows that high IU indeed negatively impacts decision-making (58), and a first study in AN shows that IU contributes to poor social problem solving (59). A number of (cognitive behavioral therapy related) interventions have been validated as successful in reducing IU across emotional disorders (60) and even in treating SE-AN (61). A first study in adolescents with AN showed reductions in IU after an adapted IU intervention (62). Further studies should be conducted to test IU interventions in adults with AN, in particular to explore how reducing IU may result in fewer emotional difficulties and improvements in quality of life and eating disorder pathology.

We argue that decision-making in severely ill patients is driven by a disturbed emotional system, disturbed allocation of reward, and altered values, eventually leading to diminished MC. Haynos and Fruzzetti (33) describe how emotion dysregulation can both be a risk factor and a maintaining factor. To further study this, we need longitudinal and experimental studies in changes over time of emotion regulation, as AN progresses and develops into SE-AN. In one longitudinal study (27) in patients with diminished MC, maladaptive decision-making remained present throughout the treatment period, independent from depression or anxiety. No studies studying the role in AN of emotional factors on MC have yet been published, but research in other areas (borderline personality disorder, depression) (63) has shown a link. Finally, further studies are needed to explore the way in which emotional dysregulation influences decision-making and relates to the development of SE-AN.

## Treatment

Therapists should be aware of emotional issues at the start of treatment and, when possible, adjust their treatment accordingly. Protocols like CBT-IU (60) or by adding Cognitive Remediation and Emotion Skills Training CREST (64) or MANTRA (65) include attention to emotional problems and IU. It may also be worthwhile to review treatments designed to address a spectrum of difficult-to-treat disorders sharing similar phenotypic and genotypic features associated with maladaptive overcontrol, such as Radically Open-Dialectical Behavior Therapy (66), for people suffering from SE-AN who are in poor physical health.

## Medico-Ethical Aspects of MC

MC can inform us about problems or erroneous decision-making in SE-AN. What to do in case of diminished MC? First and foremost, a good therapeutic relationship is wanted and needed. Even in a patient suffering from SE-AN, we need to discuss what would improve their situation, especially when starvation becomes life-threatening. One of the ultimate clinical implications of diminished MC in critically ill patients is compulsory treatment (CT). CT can be lifesaving and can also lead to positive outcomes, at least in adolescents (67). Patients generally support CT in life-threatening situations, and in their review, Elzakkers et al. (68) report that none of the studies showed a worsening in the therapeutic relationship. However, CT is not the solution for all patients with diminished MC who refuse treatment. In some situations, it may do more harm than good. Data from Denmark (69) show especially in patients with multiple prior treatments that were not separated by a period of good health CT becomes unproductive and sometimes even traumatic for the patient, increasing the likelihood of them refusing future interventions. MC ratings may be of help in the choice for CT, but the warning is, as mentioned earlier, that the MacCat-T is criticized for being too focused on cognitive and rational functioning and therefore misses the effect on decision-making of the underlying emotional dysfunction. Goldberg (70) suggests solving this by adding a narrative coherence (NC) standard to the MC ratings, that is, adding the patient's self-narrative about their illness situation. Miller Tate (71) comments on his paper by stating that SE-AN patients will easily pass this NC based on the “pathological” values that define AN and lead to an egosyntonic experience of their illness. This notion of “pathological” values complicates the discussion and concern is voiced whether the patient's autonomy with regard to MC in such a situation of starvation is not overvalued in SE-AN. By favoring autonomy over the other ethical principles (non-maleficence, beneficence, and justice) in the assessment of MC, the clinician furthermore is in danger of paying too little attention to the patient's relationships, their wishes and care needs, and long-term social context. Bloch and Green (72) propose a combination of this principle-based ethical model with care ethics, with a large role for emotions and interpersonal relationships in moral deliberation besides the issue of MC. In doing so, they underline the findings about emotional dysregulation and its effect on decision-making. In a very recent overview, Wonderlich et al. (73) point out some future directions in research and treatment of SE-AN. The first step being a better diagnostic description of SE-AN and second how best to engage and retain people with SE-AN in treatment, how to support their caretakers and tailor existing treatments or develop new ones. As we see more developments of people suffering from SE-AN being taken into hospices or palliative care, discussions about the medico-ethical aspects of this severe form of AN are needed, and protocols for clinicians, patients, and families to ensure the best interests of the patient are preserved.

## Conclusion

We need longitudinal and experimental studies in changes over time of emotion regulation, as AN progresses and develops into SE-AN. Moreover, the connection to MC in these patients has not yet been thoroughly studied in clinical research, and neither has the relationship between maladaptive decision-making, disturbed emotion processing, and MC. Finally, we think there is an urgent need for more qualitative studies in patients as well as clinicians to add to this discussion. Patient studies should aim to specifically address the issue of diminished appreciation, and clinician studies should aim to determine in more detail what it is they estimate when assessing MC.

## Author Contributions

All authors contributed equally in set up and writing.

## Conflict of Interest

The authors declare that the research was conducted in the absence of any commercial or financial relationships that could be construed as a potential conflict of interest.
